# *QuickStats: *Age-Adjusted Percentage* of Adults Aged ≥65 Years Who Have a Lot of Difficulty Hearing or Cannot Hear at All Even When Using Hearing Aids,^†^ by Urbanization Level^§^ — National Health Interview Survey, United States, 2019^¶^

**DOI:** 10.15585/mmwr.mm7033a7

**Published:** 2021-08-20

**Figure:**
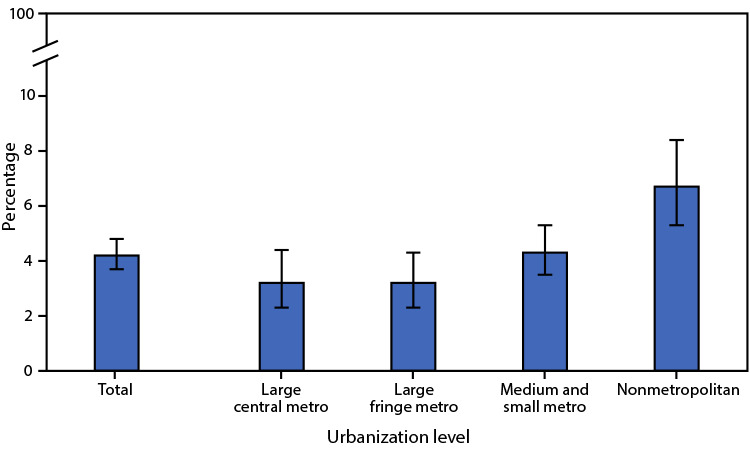
In 2019, 4.2% of U.S. adults aged ≥65 years had a lot of difficulty hearing or could not hear at all even when using hearing aids. Percentages were highest in nonmetropolitan areas (6.7%). The differences between percentages in large central (3.2%), large fringe metropolitan (3.2%), and medium and small metropolitan (4.3%) areas were not statistically significant.

**Source:** National Center for Health Statistics, National Health Interview Survey, 2019. https://www.cdc.gov/nchs/nhis.htm

